# Crocatin A: Neolignan From *Piper crocatum* Leaves Exhibited Promising Potency Against Hepatitis C Virus

**DOI:** 10.1155/tswj/6100455

**Published:** 2026-11-15

**Authors:** Tutik Sri Wahyuni, David Vincentius, Lidya Tumewu, Moi Meng Ling, Zainul Amiruddin Zakaria, Emil Salim, Achmad Fuad Hafid

**Affiliations:** ^1^ Department of Pharmaceutical Sciences Faculty of Pharmacy Universitas Airlangga Campus C Surabaya East Java Indonesia unair.ac.id; ^2^ Center for Natural Product Medicine Research and Development Institute of Tropical Disease Universitas Airlangga Surabaya East Java Indonesia unair.ac.id; ^3^ Faculty of Pharmacy Universitas Airlangga Campus C Surabaya East Java Indonesia unair.ac.id; ^4^ School of International Health Graduate School of Medicine The University of Tokyo Tokyo Japan u-tokyo.ac.jp; ^5^ Borneo Research on Algesia Inflammation and Neurodegeneration (BRAIN) Group Faculty of Medicine and Health Sciences University Malaysia Sabah Kinabalu Sabah Malaysia ums.edu.my; ^6^ Department of Chemistry Faculty of Mathematics and Natural Sciences Universitas Andalas Padang Indonesia unand.ac.id

**Keywords:** bioactivity-guided isolation, crocatin A, hepatitis C virus, molecular docking, *Piper crocatum*

## Abstract

Hepatitis C virus (HCV) is a human pathogenic virus that infects approximately 50 million people worldwide. Current therapies are costly and may lead to drug resistance during prolonged treatment, highlighting the need for new antiviral agents. *Piper crocatum* has previously been reported to have anti‐HCV activity. Accordingly, this study was aimed at isolating an active compound with anti‐HCV activity and low cytotoxicity from the 70% ethanol extract of *Piper crocatum*. Isolation was performed using a bioassay‐guided approach. Anti‐HCV activity was evaluated in Huh7it‐1 hepatocyte cells infected with the JFH1 strain. Structural elucidation of the isolated compound was carried out using ^1^H and ^13^C NMR spectroscopic analyses and comparison with published data. Bioassay‐guided isolation of the 70% ethanol extract of *Piper crocatum* leaves resulted in the isolation of crocatin A, a neolignan compound with anti‐HCV activity. The extract and crocatin A exhibited IC_50_ values of 2.29 ± 0.19 and 1.90 ± 0.51 * μ*g/mL, respectively. Both also had CC_50_ values > 100 *μ*g/mL against Huh7it‐1 hepatocytes and the Vero cell line, indicating SI > 43.67 and > 52.63, respectively. Based on these findings, the extract and crocatin A possess anti‐HCV activity while exhibiting no significant cytotoxicity toward the cell cultures used. The mode of action demonstrated stronger inhibition in the postentry step. Moreover, molecular docking was performed to predict the possible mechanism of action of the active compound, suggesting that crocatin A may interact favorably with the NS5B polymerase, potentially contributing to viral replication inhibition.

## 1. Introduction

Hepatitis C virus (HCV) is a human pathogenic virus that is transmitted through blood transfusion. According to the World Health Organization (WHO), there were around 50 million people in the world who were chronically infected with HCV in 2022 [1]. Indonesia is ranked sixth after the United States and has many patients infected with hepatitis C, with 1,400,000 people. HCV infection is considered a major public health problem because chronic hepatitis often develops into cirrhosis and hepatocellular carcinoma. In 2022, there were around 244,000 people infected with hepatitis C who died, and most of the causes were cirrhosis and hepatocellular carcinoma or primary liver cancer [1].

WHO′s recommendation for anti‐HCV is direct‐acting antivirals (DAAs) with a relatively short duration of use, namely, 12–24 weeks, depending on the presence or absence of cirrhosis [2]. The combination of DAAs with interferon‐*α* can inhibit all genotypes of the HCV with a sustained virologic response (SVR) of more than 90% and minimal side effects, but the cost is high and not affordable for all patients [3]. Only 20% of the 50 million people living with chronic hepatitis C globally have received curative DAA treatment, largely due to severe global pricing disparities and intellectual property barriers that keep these medications out of reach in developing countries [1]. In addition, there is potential for resistance in patients if antiviral therapy is carried out in the long term [4]. Thus, efforts to find new drugs, especially for complementary and/or alternative purposes, are still ongoing from a clinical and economic perspective. Medicinal plants are provided to support liver health, impede viral entry, and target viral replication when access to conventional drugs is limited [5].

Several plants from the Piperaceae family have been reported to have anti‐HCV activity, such as *Piper cubeba* and *Piper nigrum* L. [6]. The 96% ethanol extract of *Piper betle* leaves is reported to be an anti‐HCV substance [7]. *Piper crocatum*, with the synonym *Piper crocatum* Ruiz and Pav. known as red betel, is a medicinal plant in Indonesia [8]. Li et al. [9] have isolated compounds from the leaves of *Piper crocatum* Ruiz and Pav. and obtained 24 compounds belonging to the phenolic, monoterpene, sesquiterpene, phenolic amide glycosides, neolignans, and flavonoid C‐glycoside groups. Information on the anti‐HCV activity and the compounds responsible from the leaves of *Piper crocatum* Ruiz and Pav. plant still needs to be studied. It was also reported that lignans and neolignans from the genus *Piper* possess anti‐HCV activities as multitarget inhibitors, blocking viral proteins including NS5B polymerase [10].

In this study, we used an in vitro research model in the form of Huh7it‐1 cell culture and Japan fulminant hepatitis 1 (JFH1) virus, which is an HCV with genotype 2a, to determine the activity of the 70% ethanol extract of *Piper crocatum* leaves, fractions, and isolated compounds. The isolation of the compound was conducted by bioassay‐guided isolation to obtain an active anti‐HCV compound. Further evaluation was conducted by molecular docking assay to predict the mechanism of action of the active compound. The HCV NS3 protease and NS5B polymerase were selected as molecular targets due to their critical roles in the HCV replication cycle.

## 2. Materials and Methods

### 2.1. Plant Material

*Piper crocatum* leaves were obtained from Jombang City, East Java, Indonesia. Dr. Ratih Damayanti, S.Hut., M.Si., Directorate of Scientific Collection Management at BRIN Cibinong, Jakarta, Indonesia, determined the plants for identification and authentication. The plant determination letter (B‐2954/II.6.2/IR.01.02/8/2024) was issued by the Directorate of Scientific Collection Management at BRIN Cibinong, Jakarta, Indonesia, and indicates that the plants used are *Piper crocatum*.

### 2.2. Extraction and Isolation

*Piper crocatum* leaves were dried at room temperature and powdered. They were then extracted using 70% ethanol at room temperature for 24 h. The extract was then filtered, and the residue was re‐extracted using 70% ethanol. The entire filtrate was concentrated using an evaporator at a temperature not exceeding 40°C, and a 70% ethanol extract of *Piper crocatum* leaves was obtained. The 70% ethanol extract was fractionated using vacuum liquid chromatography (VLC) with various concentrations of mobile phase (n‐hexane:dichloromethane, dichloromethane:ethyl acetate, and ethyl acetate:methanol) to obtain nine fractions (F1–F9). Only six fractions (F1, F2, Fraction 4 [F4], F6, F8, and F9) showed an inhibition percentage of more than 80% in anti‐HCV screening. Furthermore, the IC_50_ value of each active fraction was determined; F4 was selected for purification using high‐performance liquid chromatography (HPLC) with an isocratic methanol–water solvent system (7:3 *v*/*v*); Zorbax Eclipse XDB‐C18 column (9.4 × 250 mm, 5 *μ*m), flow rate 1.0 mL/min; ultraviolet (UV) detection at 210 nm to obtain five fractions (F4.1–F4.5). Fraction 4.2 was identified as Compound (1).

### 2.3. Identification of Compound (1)

Fraction F4.2 was first checked for its purity using thin‐layer chromatography (TLC) in two pairs of stationary phase and mobile phase, namely, Condition A and Condition B. Condition A was used silica gel 60 F_254_ as stationaty phase (Merck) with mobile phase methanol:water (1:2 *v*/*v*), while condition B was used silica gel RP‐18 F_254_ stationary phase (Merck) with mobile phase methanol:air (3:1 *v*/*v*). Further structure identification was conducted using spectroscopic data, including ^1^H (400 MHz) and ^13^C (100 MHz) NMR (JEOL ECS‐400), and the data were compared with the reference.

### 2.4. Cell Culture and Virus Preparation

Huh7it‐1 hepatocyte cells were cultured in Dulbecco′s Modified Eagle Medium (GIBCO Invitrogen, Carlsbad, CS, United States) and supplemented with 10% fetal bovine serum (Biowest, Nualle, France) and 0.1% penicillin–streptomycin solution (SIGMA) at 5% CO_2_ and 37°C. JFH1 virus was developed from a patient infected with hepatitis C and propagated on Days 3, 4, and 7, followed by titer examination every day [11].

### 2.5. Anti‐HCV Activity Test

The extract, fractions, and Compound (1) from *Piper crocatum* were determined for their anti‐HCV activity. The activity test began with seeding Huh7it hepatocyte cells at a density of 5.6 × 10^4^ cells/well in a 48‐well plate and incubating for 24 h at 37°C with 5% CO_2_. Each test sample (extract, fractions, and Compound (1)) at concentrations of 0.01, 0.1, 1, 10, 50, and 100 *μ*g/mL was mixed with JFH1 virus at a multiplicity of infection of 0.1 (MOI 0.1). The test samples were inoculated into cells in each well in a 48‐well plate, and the cells were incubated for 2 h to allow the virus to be absorbed into the cells. After 2 h, the supernatant was discarded, replaced with medium, and incubated again for 48 h. After 48 h, the supernatant was taken, and virus titration and basic immunostaining were carried out to determine the percentage inhibition value of the sample [12, 13]. The IC_50_ value was determined by probit analysis using IBM SPSS Statistics software 25.0. Further studies were conducted to determine the mode of action assay to examine the activity only in the entry step or postentry step. The infected cells were treated for 2 h during virus inoculation or after inoculation at a concentration of 30 *μ*g/mL, and the percentage inhibition was determined.

### 2.6. Cytotoxicity Test

A cytotoxicity test was carried out on the extract, fractions, and Compound (1) using MTT reagent (3‐(4,5‐dimethylthiazol‐2‐yl)‐5‐(3‐carboxymethoxyphenyl)‐2‐(4‐sulfophenyl)‐2H‐tetrazolium) (Thermo Fisher). Huh7it cells were exposed to test samples at various concentrations (0.01, 0.1, 1, 10, 50, and 100 *μ*g/mL) and incubated for 48 h. After that, the existing medium was discarded and replaced with MTT reagent and then incubated for 4 h. To dissolve the formazan formed, DMSO was added. Absorbance was detected at wavelengths of 560 and 750 nm using a microplate multidetector reader (Sigma). The absorbance of the sample was detected by comparing it with the control [14]. Moreover, a further assay was done by evaluating the cytotoxicity effect in Vero cells, a normal cell line, using the same method. The CC_50_ value was determined by probit analysis using IBM SPSS Statistics software 25.0.

### 2.7. Molecular Docking Analysis of Crocatin A Binding Affinity Toward NS3 and NS5B Proteins

The binding affinity of crocatin A toward the HCV target proteins, NS3 protease and NS5B polymerase, was evaluated through molecular docking studies. The 3D crystallographic structure of the target proteins was downloaded from the Protein Data Bank using PDB IDs 4OK5 for NS3 protease and 3FQL for NS5B polymerase. Protein preparation was carried out by removing water molecules and cocrystallized ligands, followed by the addition of polar hydrogens and assignment of Kollman charges using AutoDock Tools [15]. The structure of crocatin A was prepared and energy‐minimized prior to docking analysis. Molecular docking simulations were subsequently performed using AutoDock 1.5.7 and Open Babel, followed by conversion into .pdb and .pdbqt file formats [16]. The molecular docking protocol was adapted from Kamboj et al. [17], whereas the grid box parameters were independently determined based on the position of the cocrystallized ligand in each protein structure. For PDB ID 4OK5, the grid box was centered at (−19.170, −31.489, and 31.798) with dimensions of 50 × 50 × 50 Å along the x, y, and z axes, whereas for PDB ID 3FQL, the grid box was centered at (−13.390, −13.724, and 9.880) with dimensions of 40 × 40 × 40 Å. Validation of the docking protocol by redocking the native ligand yielded RMSD values of 0.877 Å for 4OK5 and 0.024 Å for 3FQL. The docking results were analyzed to determine binding affinity, types of molecular interactions, and amino acid residues involved in ligand binding. Molecular interactions and residue contacts, including their position and interaction distances, were visualized using BIOVIA Discovery Studio Visualizer (BIOVIA and Dassault Systemes) and LigPlot+ [18].

## 3. Result and Discussion

### 3.1. Extraction and Isolation

*Piper crocatum* leaves (228 g) were extracted using 70% ethanol as a solvent, and 26.22 g of extract was obtained (% yield: 11.5%). The extract (25 g) was then subjected to VLC and yielded nine fractions (F1–F9) as shown in Table 1. F4 (73.8 mg) was further purified by preparative HPLC to obtain Compound (1) (8.2 mg; % yield: 0.003%). The percentage yield obtained from the extraction process is related to the amount of bioactive compounds contained. The higher the percentage yield indicated, the more substances are extracted from the raw material. The resulting extract was then subjected to antiviral activity testing against HCV and cytotoxicity assays. The isolation of Compound (1) was based on bioassay‐guided isolation, which means that purification was conducted on the active fraction. Moreover, the amount and the TLC profile of the active fraction should also be taken into consideration.

**Table 1:** The weight of nine fractions from the 70% ethanol extract of *Piper crocatum*.

Fraction groups	Fraction weights (mg)
Fraction 1 (F1)	35.4
Fraction 2 (F2)	6.8
Fraction 3 (F3)	13.8
Fraction 4 (F4)	73.8
Fraction 5 (F5)	57.0
Fraction 6 (F6)	13.9
Fraction 7 (F7)	1.9
Fraction 8 (F8)	101.8
Fraction 9 (F9)	872.4

The visualization of compounds separated on the stationary phase using UV wavelengths ranging from 200 to 400 nm is referred to as quenching [19]. Compounds that absorb radiation under UV exposure at 254 nm typically exhibit conjugated double‐bond characteristics, such as flavonoids, polyphenols, coumarins, anthraglycosides, and certain types of alkaloids (quinoline, isoquinoline, and indole). The F4 and F4.1–F4.5 showed conjugated double‐bond characteristics of compounds under UV 254.

The results of F4.2 TLC can be observed in Figure 1. Identification shows a single dominant black spot with Rf = 0.63 when visualized using UV light at a wavelength of 254 nm, with a stationary phase of silica gel F_254_ and a mobile phase of n‐hexane:ethyl acetate (1:2 *v*/*v*). A single dominant black spot was observed with Rf = 0.37 when visualized using UV light at a wavelength of 254 nm with a stationary phase of silica gel RP‐18 F_254_ and a mobile phase of methanol:water (3:1 *v*/*v*). The results indicated that F4.2 was a pure compound and was further identified as Compound (1).

**Figure 1:**
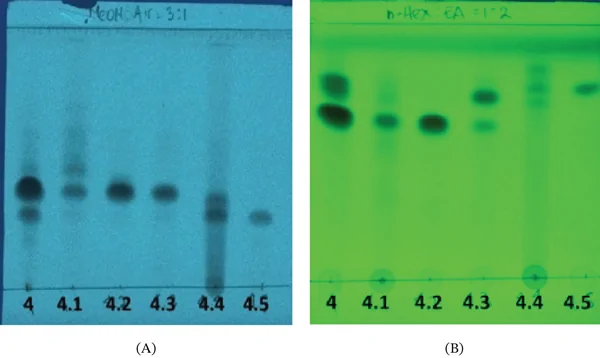
Thin‐layer chromatograms of each subfraction (F4.1–F4.5) from Fraction 4 (F4) of the 70% ethanol extract of *Piper crocatum* leaves under (A) Condition A and (B) Condition B at a wavelength of 254 nm.

According to studies conducted by Li et al. [20], there are 12 active compounds isolated from *Piper crocatum*. These compounds were identified as (1) pipercroside A, (2) pipercroside B, (3) 2,5‐dimethoxy‐3‐glucopyranosylcinnamic alcohol, (4) cimidahurinin, (5) erigeside II, (6) syringin, (7) *β*‐phenylethyl *β*‐D‐glucoside, (8) methyl salicylate 2‐*O*‐*β*‐D‐glucopyranoside, (9) icariside D1, (10) 4‐hydroxybenzoic acid *β*‐D‐glucosyl ester, (11) benzyl *β*‐D‐glucoside, and (12) phenylmethyl 6‐*O*‐*α*‐L‐arabinofuranosyl‐*β*‐D‐glucopyranoside.

The identification of Compound (1) from F4 of the 70% ethanol extract of *Piper crocatum* is consistent with previous phytochemical investigations that reported the presence of multiple glycosidic and phenolic constituents in this species. As demonstrated by Li et al. [20], 12 active compounds were successfully isolated from *Piper crocatum*, including pipercroside A and B, syringin, and icariside D1, among others. The overlap between the current findings and earlier reports suggests that the bioactive profile of *Piper crocatum* is largely attributable to these structurally diverse metabolites.

The presence of glycosides such as pipercroside A and B, together with phenolic derivatives like syringin and methyl salicylate glucoside, provides a strong chemical basis for the biological activities observed in the crude extract and its fractions [20]. These compounds are known to exhibit antioxidant, anti‐inflammatory, and antiviral properties [21], which may explain the significant anti‐HCV activity detected in the present study. Furthermore, the isolation of Compound (1) as a pure metabolite from F4 contributes directly to the antiviral potential of *Piper crocatum*, whereas synergistic interactions among the 12 identified compounds may further enhance its overall efficacy.

### 3.2. Structural Elucidation of Compound (1)

Based on the ^1^H NMR spectrum (400 MHz, CDCl_3_), there are three methoxy groups bound to aromatics [*δ*_H_ 3.78 (6H, s) and 3.77 (3H, s)]. The chemical shift *δ*_H_ 3.78 indicates the symmetric position of the methoxy group bound to aromatics at C‐3 (3‐OCH_3_) and C‐5 (5‐OCH_3_). Other signals indicate the presence of methoxy groups attached to carbon [*δ*_H_ 3.68 (3H, s) and 3.29 (3H, s)], methyl attached to aliphatic carbon [*δ*_H_ 1.28 (3H, d, *J* = 7.6 Hz)], allyl groups [*δ*_H_ 5.83 (1H, m), 5.23 (1H, d, *J* = 4.0 Hz), 5.20 (1H, s), 2.46 (1H, m), 2.35 (1H, m)], aromatic protons C‐2/C‐6 [*δ*_H_ 6.25 (2H, s)], olefinic protons part of the enone system [*δ*_H_ 6.17 (1H, s)], benzylic methine protons [*δ*_H_ 3.33 (1H, d, *J* = 6.4 Hz)], methine protons [*δ*_H_ 2.30 (1H, m)], and carbinol proton [*δ*_H_ 5.35 (1H, s)].

The ^13^C NMR spectrum indicated the presence of a ketone group (*δ*_C_ 191.6), an ester carbonyl (*δ*_C_ 169.5), three methoxy groups attached to aromatics (*δ*_C_ 60.8 and 55.9), a 3,4,5‐trimethoxyphenyl ring (*δ*_C_ 152.9, 137.1, 133.1, and 105.9), a carbinol carbon (*δ*_C_ 78.7), two methoxy groups attached to an aliphatic carbon (*δ*_C_ 55.5 and 55.1), a methyl group (*δ*_C_ 17.3), an allyl group (*δ*_C_ 133.8, 119.5, and 35.1), a methyl group attached to a carbonyl (*δ*_C_ 21.1), an olefinic carbon (*δ*_C_ 152.6 and 124.5), and two quaternary carbons (*δ*_C_ 94.8 and 48.1). This spectroscopic data was used to deduce the core structure as a bicyclo[3,2,1]octane derivative. The ^1^H NMR chemical shift of the methyl group attached to C‐8 [*δ*_H_ 1.28 (3H, d, *J* = 7.6 Hz)] indicated a *trans*‐relationship between the C‐7 [*δ*_H_ 3.33 (1H, d, *J* = 6.4 Hz)] aryl and C‐8 methyl substituent. On comparison of the above data with the reference, the structure of Compound (1) was determined as crocatin A [22]. The ^1^H and ^13^C NMR of Compound (1) compared to the reference are shown in Table 2. The chemical structure of Compound (1) is shown in Figure 2.

**Table 2:** ^1^H (400 MHz) and ^13^C (100 MHz) NMR data of Compound (1) in CDCl_3._

Position	^1^H NMR		^13^C NMR	
	*δ*_H_	*δ*_H_ ref	*δ*_C_	*δ*_C_ ref
1			133.1	133.0
2	6.25 (2H, s)	6.26 (2H, s)	105.9	105.9
3			152.9	152.8
4			137.1	137.1
5			152.9	152.8
6	6.25 (2H, s)	6.26 (2H, s)	105.9	105.9
7	3.33 (1H, d, *J* = 6.4 Hz)	3.33 (1H, d, *J* = 6.5 Hz)	59.7	59.6
8	2.30 (1H, m)	2.30 (1H, m)	48.9	48.8
9	1.28 (3H, d, *J* = 7.6 Hz)	1.28 (3H, d, *J* = 7.0 Hz)	17.3	17.2
1 ^′^			48.1	48.0
2 ^′^	5.35 (1H, s)	5.35 (1H, s)	78.7	78.7
3 ^′^			94.8	94.7
4 ^′^			191.6	191.5
5 ^′^			152.6	152.6
6 ^′^	6.17 (1H, s)	6.17 (1H, s)	124.5	124.5
7 ^′^	2.46 (1H, m); 2.35 (1H, m)	2.46 (1H, m); 2.34 (1H, m)	35.1	35.1
8 ^′^	5.83 (1H, m)	5.83 (1H, m)	133.8	133.7
9 ^′^	5.23 (1H, d, *J* = 4.0 Hz); 5.20 (1H, s)	5.23 (1H, d, *J* = 4.0 Hz); 5.20 (1H, s)	119.5	119.4
COO			169.5	169.4
3‐OCH_3_	3.78 (6H, s)	3.78 (6H, s)	55.9	55.8
4‐OCH_3_	3.77 (3H, s)	3.77 (3H, s)	60.8	60.7
5‐OCH_3_	3.78 (6H, s)	3.78 (6H, s)	55.9	55.8
3 ^′^‐OCH_3_	3.68 (3H, s)	3.68 (3H, s)	55.5	55.4
5 ^′^‐OCH_3_	3.29 (3H, s)	3.29 (3H, s)	55.1	55.0
COOCH_3_	2.25 (3H, s)	2.25 (3H, s)	21.1	21.0

*Note:* Ref.: Arbain et al. [22] (CDCl_3_, ^1^H 500 MHz, ^13^C 125 MHz).

**Figure 2:**
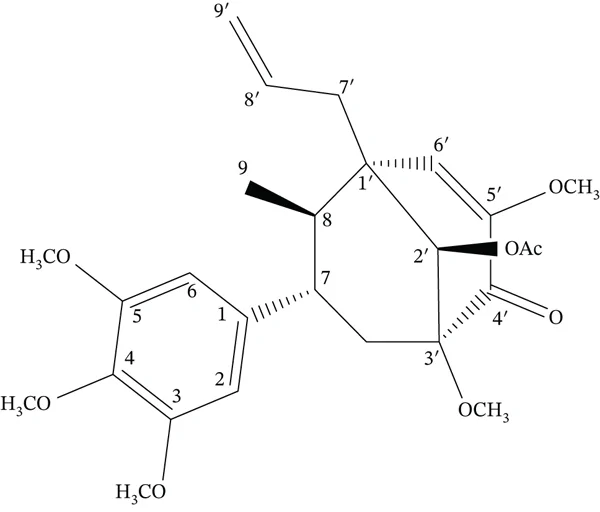
Chemical structure of Compound (1).

Crocatin A represents a structurally unique metabolite within *Piper crocatum*. Crocatin A is a neolignan compound. Neolignans are plant secondary metabolites derived from the oxidative coupling of phenylpropanoids. The presence of multiple methoxy substituents and conjugated systems suggests potential biological activities, particularly antioxidant and antiviral properties, which are commonly associated with phenolic and enone‐containing compounds [23]. The isolation of crocatin A as a pure metabolite provides a direct link between chemical structure and biological activity [22]. As demonstrated in the antiviral assays, crocatin A exhibited significant anti‐HCV activity with minimal cytotoxicity, highlighting its potential as a lead compound for drug development. The structural features identified through NMR analysis, such as the trimethoxyphenyl ring and enone system, may be critical determinants of its antiviral mechanism, possibly through modulation of viral replication pathways or interaction with viral enzymes. One representative of other neolignans is silymarin flavonolignan, which was isolated from the seeds of *Silybum marianum*, and it is the most commonly consumed herbal product among HCV‐infected patients in Western countries. It has been shown to block HCV production and increase anti‐inflammatory and antiproliferative gene expressions without affecting serum albumin levels in the clinical phase [24, 25].

### 3.3. Anti‐HCV Activity and Cytotoxicity of 70% Ethanol Extract, Fractions, and Compound (1)

The percentage inhibition of the extract, selected fractions, and compound at the tested concentrations is shown in Figure 3. The cytotoxicity assay demonstrated no toxic effect, with a percentage cell viability > 90% for all concentrations tested. Moreover, the IC_50_ and CC_50_ values are presented in Table 3. The 70% ethanol extract of *Piper crocatum* leaves exhibited an IC_50_ value of 2.29 ± 0.19 * μ*g/mL. According to Indrayanto et al. [26], extracts derived from natural materials that have an IC_50_ value of < 10 *μ*g/mL are considered to possess good biological activity. Furthermore, Versiaty et al. [23] emphasized that both extracts and fractions originating from natural sources can be categorized as active anti‐HCV agents if their IC_50_ value is < 30 *μ*g/mL. In line with these criteria, the 70% ethanol extract of *Piper crocatum* is classified as active because it has an IC_50_ value of < 30 *μ*g/mL.

**Figure 3:**
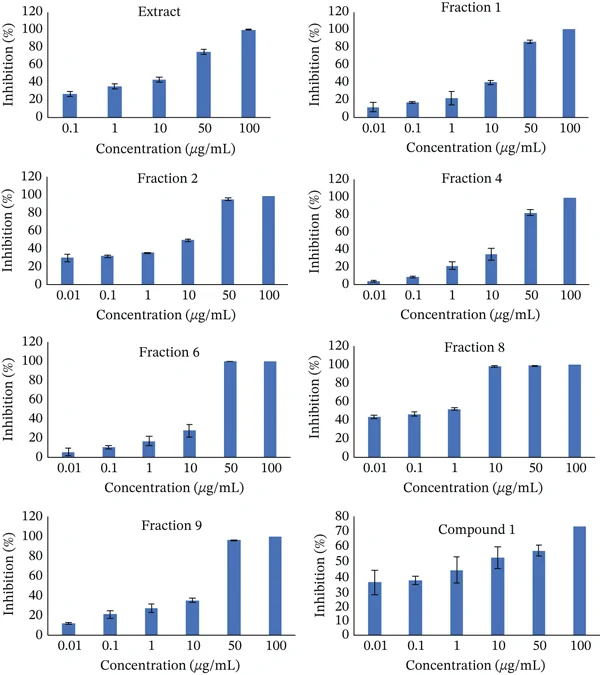
Dose‐dependent inhibition of HCV infection by ethanol extract, F1, F2, F4, F6, F8, F9, and Compound (1) from *Piper crocatum*. Data represent the mean ± SD from three replicate independent experiments.

**Table 3:** Anti‐HCV activity (IC_50_) against JFH1 virus and cytotoxicity (CC_50_) against Huh7it cells of the extract, fractions, and Compound (1).

Samples	IC_50_^a^ (*μ*g/mL)	CC_50_^a^ (*μ*g/mL)	SI
Extract	2.29 ± 0.19	> 100	> 43.67
F1	3.37 ± 0.52	> 100	> 29.67
F2	0.81 ± 0.23	> 100	> 123.46
F4	5.90 ± 0.58	**> 100**	> 16.95
F6	4.60 ± 0.60	> 100	> 21.74
F8	0.08 ± 0.01	> 100	> 1250
F9	2.32 ± 0.29	> 100	> 43.10
Compound (1)	1.90 ± 0.51	> 100	> 52.63

^a^Data represent the mean ± SD of data from three independent experiments.

In addition to the crude extract, all tested fractions, namely, fractions F1, F2, F4, F6, F8, and F9, also exhibited an IC_50_ value < 30 *μ*g/mL. The activities across multiple fractions suggest that the bioactive constituents responsible for the antiviral effect are distributed throughout different polarity ranges of the fractions rather than being confined to a single fraction. Such a distribution pattern indicates the presence of diverse phytochemical groups contributing to its anti‐HCV activity. The results not only reinforce the classification of these fractions as active against HCV but also provide a strong rationale for subsequent isolation and structural elucidation of the active compounds.

Compound (1) had an IC_50_ value of 1.90 ± 0.51 * μ*g/mL (or equivalent to 4.12 ± 1.10 * μ*M), so it can be categorized as active as an anti‐HCV due to the IC_50_ value being less than 30 *μ*g/mL. Compound (1), which was identified as crocatin A, was reported for the first time to have activity as an anti‐HCV. In addition to its antiviral potency, crocatin A demonstrated a CC_50_ value of > 100 *μ*g/mL when tested against Huh7it cells, indicating that the compound is noncytotoxic under the experimental conditions employed. Moreover, no cytotoxic effect was also confirmed with Vero cells and demonstrated CC_50_ > 100 *μ*g/mL. This suggests that crocatin A possesses antiviral activity without adversely affecting host cell viability. The selectivity index (SI) value obtained from Compound (1) is > 52.63, indicating that the compound is worthy of further testing as a candidate for anti‐HCV. In general, a standard SI value > 10 in natural material samples can be the basis for determining the feasibility of further testing [26].

The mode of action analysis showed inhibition of 34.0*%* ± 3.12*%* and 62.9*%* ± 1.56*%*, respectively, at entry and postentry steps for a concentration of 30 *μ*g/mL. These indicated dominant action in the postentry step, which includes the replication process. Many current treatments use DAAs that inhibit postentry, especially in the replication steps. One of them is sofosbuvir, an NS5B inhibitor, which possesses an IC_50_ value of 0.06 ± 1.76 * μ*M [27].

### 3.4. Predicted Binding Affinity of Crocatin A Toward HCV NS3 Protease and NS5B Polymerase Through Molecular Docking

An in silico assay was performed to predict the potential interaction of crocatin A with the HCV proteins NS3 protease and NS5B polymerase. The molecular docking results showed that crocatin A exhibited a favorable binding affinity toward NS5B polymerase (PDB ID: 3FQL), with a binding energy of −9.27 kcal/mol, comparable to that of the reference drug sofosbuvir (−9.21 kcal/mol). In contrast, crocatin A displayed a lower binding affinity toward NS3 (PDB ID: 4OK5), with a binding energy of −6.30 kcal/mol (Figure 4). These results indicate that crocatin A has a stronger predicted interaction with NS5B than with NS3 under the present docking conditions. However, the interacting residues identified for crocatin A differ from those typically associated with the nucleotide‐binding catalytic site occupied by sofosbuvir, suggesting that crocatin A may bind to a distinct region of NS5B. Therefore, the comparable binding energies should not be interpreted as evidence of an identical binding mode or inhibitory mechanism, and further structural and biochemical studies are required to confirm its binding site and mode of action.

**Figure 4:**
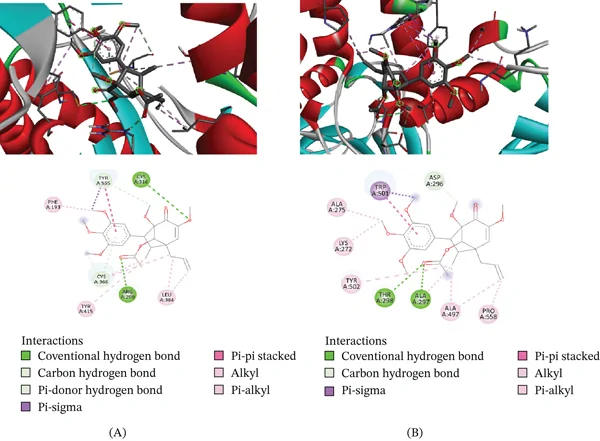
Visualization of the 3D (three‐dimensional) and 2D (two‐dimensional) molecular docking interaction of crocatin A with (A) NS5B Protein (PDB ID 3FQL) and (B) NS3 Protein (PDB ID: 4OK5).

The findings indicated that crocatin A may exhibit anti‐HCV potential, which could be associated with its predicted binding interaction with the HCV NS5B polymerase.

## 4. Conclusion

Crocatin A was isolated from the 70% ethanol extract of *Piper crocatum* leaves and demonstrated anti‐HCV activity with an IC_50_ value of 3.83 ± 3.59 * μ*g/mL. In addition, crocatin A shows no significant cytotoxicity, with a CC_50_ value greater than 100 *μ*g/mL. Molecular docking analysis predicted that crocatin A may interact with the HCV NS5B polymerase, suggesting its potential inhibitory activity against viral replication. These results indicate that crocatin A is a potential lead compound that warrants further investigation for the development of anti‐HCV agents.

## Funding

This study was funded by the Indonesian Ministry of Research, Education, Science, and Technology through the grant scheme of the Fundamental Research Program with Contract Number 059/C3/DT.05.00/PL/2025.

## Conflicts of Interest

The authors declare no conflicts of interest.

## Data Availability

The data that support the findings of this study are available upon request from the corresponding author. The data are not publicly available due to privacy or ethical restrictions.
